# Changes in Phenylpropanoid and Trichothecene Production by *Fusarium culmorum* and *F. graminearum* Sensu Stricto via Exposure to Flavonoids

**DOI:** 10.3390/toxins10030110

**Published:** 2018-03-05

**Authors:** Katarzyna Bilska, Kinga Stuper-Szablewska, Tomasz Kulik, Maciej Buśko, Dariusz Załuski, Sebastian Jurczak, Juliusz Perkowski

**Affiliations:** 1 Department of Microbiology and Mycology, University of Warmia and Mazury in Olsztyn, Oczapowskiego 1A, 10-719 Olsztyn, Poland; tomaszkulik76@gmail.com (T.K.); seba.jurczak@gmail.com (S.J.); 2 Department of Chemistry, Poznan University of Life Sciences, Wojska Polskiego 75, 60-637 Poznan, Poland; kstuper@up.poznan.pl (K.S.-S.); mabu@up.poznan.pl (M.B.); julperk@up.poznan.pl (J.P.); 3 Department of Plant Breeding and Seed Production, University of Warmia and Mazury in Olsztyn, Plac Łódzki 3, 10-727 Olsztyn, Poland; dariusz.zaluski@uwm.edu.pl

**Keywords:** *Fusarium*, flavonoids, naringenin, quercetin, kaempferol, apigenin, luteolin, trichothecenes

## Abstract

Flavonoids are a group of hydroxylated polyphenolic compounds widely distributed in the plant kingdom. Biosynthesis of these compounds involves type III PKSs, whose presence has been recently predicted in some fungal species through genome sequencing efforts. In this study, for the first time it was found that Fusaria produce flavonoids on solid YES medium. Naringenin, as the central precursor of all flavonoids, was produced at highest quantities, followed by quercetin, kaempferol, apigenin and luteolin. In plants, flavonoids are involved in the protection of cereals to a wide range of stresses, including host defense against Fusaria. Under in vitro conditions, strains of *Fusarium culmorum* and *F. graminearum* sensu stricto were incubated at levels of flavonoids close to amounts produced by cereals in response to fungal infection. The amounts of exogenous naringenin, apigenin, luteolin, kaempferol and quercetin were reduced and converted by fungi to the other flavonoid derivatives. Treatment of fungi with naringenin derivatives led to the inhibition of naringenin production. Correspondingly, the production of fungal-derived phenolic acids decreased in flavonoid treated samples, although this effect appeared to be dependent on the strain, flavonoid molecule and its concentration. Fusaria showed high variability in trichothecene production in response to flavonoids. With emphasis on quercetin, mycotoxin accumulation in the media was significantly decreased by luteolin, kaempferol, naringenin and apigenin. However, in some cases, apigenin led to the increase of mycotoxin content in the media. Gene expression experiments of *Tri* genes responsible for trichothecene biosynthesis (*Tri4*, *Tri5* and *Tri10*) proved that the inhibition of mycotoxin production by flavonoids occurred at the transcriptional level. However, the changes in *Tri* transcript levels were not significant in most apigenin and all kaempferol-treated cultures. In this study, a link was established between antioxidant and antiradical properties of flavonoids and their effects on fungi.

## 1. Introduction

The genus *Fusarium* encompasses a wide diversity of fungal pathogens of high agricultural, economic and medical importance [1]. In cereal production, Fusaria cause severe diseases associated with significant yield losses, including root and crown rot, Fusarium head blight (FHB) in barley and wheat and Fusarium ear rot (FER) in maize. In addition to reducing yields, Fusaria also cause quality deterioration by contamination of the grain with mycotoxins such as trichothecenes, which are a major health concern for humans and animals [2].

Synthetic fungicides, principally from the azole class, play a prominent role in controlling Fusaria in the field. However, the efficacy of azoles for disease control often remains contradictory, presumably due to the difficulty of timing and targeting fungicides to heads [3] and the apparently increased resistance of fungi to this fungicide class [4]. In addition, azole residues can disperse and persist in the environment due to repeated use of these fungicides [5,6,7], which have a considerable impact on ecosystem health and functionality [8]. 

The risk of human health and the environment has been lately of significant concern. In order to face it the intensive research into development of novel and environmentally friendly disease management strategies has been proposed [9]. Previous studies have indicated that plant-derived phenylpropanoids may represent a break-through in the protection of crops against fungal pathogens [10,11]. This group of plant secondary metabolites is synthesized through the phenylpropanoid pathway and can be divided into two groups: Flavonoid phenylpropanoids (flavones, flavonols, flavanols, and flavanones) and non-flavonoid phenylpropanoids (stilbenes, lignans and phenolic acids) [12]. The first group encompasses nearly one hundred putative flavonoids which have been identified through metabolomic approaches to play a role in resistance mechanisms against Fusaria. Many of these metabolites correspond to glucoside derivatives of kaempferol and quercetin belonging to the flavonol class. In addition, several compounds of the flavanol, flavanone (naringenin), flavone (apigenin and vitexin derivatives) and anthocyanin classes were also highlighted [13]. 

The role of flavonoids in plant defense has been previously reviewed in Treutter [14,15], Mierziak et al. [16], Gauthier et al. [13] and Atanasova-Penichon et al. [12]. Most of these compounds are bioactive and contribute to defense mechanisms against abiotic and biotic stresses, environmental interactions [17,18] and play an important role in physiological regulations and signaling [19]. While the production of these secondary metabolites has been well documented in plants, much less is known about the biosynthesis of these compounds by fungi including plant pathogenic species [20].

**Figure 1:**
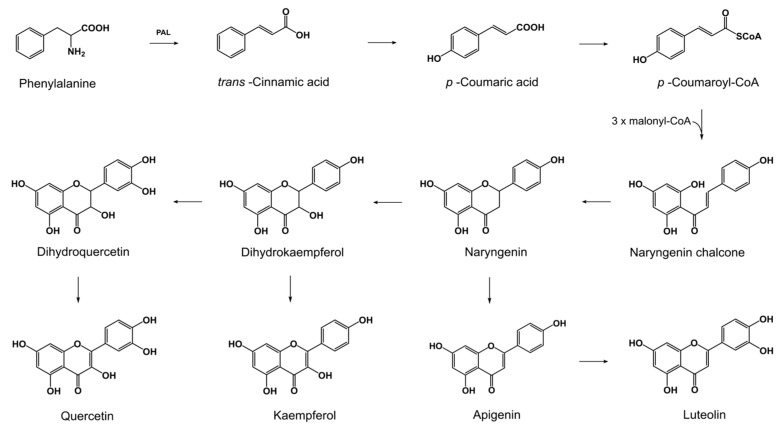
Schematic representation of biosynthesis of selected phenylpropanoids.

Biosynthesis of flavonoids involves diverse enzymes with specialized activities [21,22]. The first committed step of this pathway is catalyzed by chalcone synthase, belonging to well-studied plant type III PKS. The process begins with the sequential condensation of three acetate units from malonyl-CoA into a 4-coumaroyl-CoA molecule. Subsequently, cyclization reaction leads to the formation of an aromatic tetraketide, naringenin chalcone [23] (Figure 1). Although type III PKSs are widely studied in plants, their presence in some fungal species has mainly been predicted through recent fungal genome sequencing efforts [23,24]. Limited gene expression studies supported the functionality of the putative type III PKSs in *Aspergillus oryzae* [25]. Hypothetical chalcone synthase of *F. graminearum* reference PH-1 strain (NRRL 31084) is available in the GenBank database [26] under accession number XP_011320399, suggesting that the production of flavonoids can be widespread in the genus *Fusarium*. 

The main mechanism of action of flavonoids results from their antioxidant properties [27,28,29,30], owing to prevention or quenching of reactive oxygen species (ROS), generated by both the pathogen and the plant during infection. The other roles of flavonoids in plant defense are associated with reinforcement of cell walls restricting pathogen access to nutrients [14,15] and the inhibition of the activity of plant cell wall degrading enzymes secreted by fungi [14,15]. Limited in vitro studies showed that flavonoids display molecule-dependent growth inhibitory effects on fungi [31,32], including *Fusarium culmorum* and *F. graminearum* sensu stricto (s.s.) [33,34,35]. However, their growth inhibitory effect on Fusaria appears to be weaker than phenolic acids [12]. 

Limited studies also indicated that flavonoids appear to interact directly with fungal secondary metabolism. Flavonoids have been shown to exert an inhibitory effect on mycotoxin production by fungi such as aflatoxin [36,37] and patulin production [38]. Inhibition of trichothecene biosynthesis by flavonoids was revealed by Desjardins et al. [39] and Takahashi-Ando et al. [40], who showed an inhibitory effect of flavones on cytochrome P450 monooxygenase-catalyzing conversion of trichodiene (the first chemical intermediate in trichothecene biosynthesis) to oxygenated trichothecenes [13]. A more recent study by Bollina and Kushalappa [35] showed that other flavonoids (naringenin and quercetin) completely suppress trichothecene production of a single *F. graminearum* strain at early stages of incubation on artificial media. 

The main purpose of our research was to study the changes in phenylpropanoid (flavonoid and phenolic acid) and trichothecene profiles via exposure to flavonoids. To achieve it, different strains of *F. culmorum* and *F. graminearum* s.s. were incubated in the presence of different flavonoid aglycones: naringenin, apigenin, luteolin, kaempferol and quercetin. These compounds were selected because of their contribution to resistance to Fusaria in cereals [34,41,42] and their well-known antioxidant properties [29,30]. Concentrations of flavonoids close to the amounts quantified in kernels after *Fusarium* infection were also used [43]. The effect of treatment with these compounds was assessed on fungal secondary metabolic profiles. It was found that exogenous flavonoids affected the phenylpropanoid profiles of fungi. Production of flavonoids by Fusaria was also revealed here for the first time. The impact of exogenous flavonoids on both trichothecene accumulation in the media and the expression of *Tri* genes involved in trichothecene biosynthesis were also analyzed. An attempt was made to establish a link between the antioxidant and antiradical properties of flavonoids and their effects on fungi.

## 2. Results and Discussion

### 2.1. Production of Flavonoids by Fusaria

Previous in vitro studies showed that different strains of *F. culmorum* and *F. graminearum* s.s. accumulate phenolic acids in the media [44,45]. In this study, we screened fungi for flavonoid production. We found that besides phenolic acids, YES + fungal controls exhibited increased accumulation of flavonoids (Supplementary File S1). Naringenin, as the central precursor of the other flavonoids [46], was quantified at the highest levels (comprising 25.8–28.4% of the flavonoids produced by Fusaria). In plants, naringenin can be converted to apigenin by flavone synthase [47] (Figure 1). Apigenin can be further hydroxylated by flavonoid 3′ hydroxylase to form luteolin [48]. The presence of both flavones has been detected in fungal cultures. Apigenin comprised 15.9–17.9%, while luteolin reached 9.5–12.2% of flavonoids. Besides flavones, Fusaria produced flavonols (kaempferol and quercetin), which were quantified at higher levels than the first of the aforementioned class of flavonoids. Kaempferol, which is converted from naringenin by flavonol synthase [22], reached 20.7–22.2%. Quercetin, which is synthesized from kaempferol, yielded 21.9–25.3% of flavonoids. In plants, the formation of quercetin involved cytochrome P450 flavonoid monooxygenase associated to a cytochrome P450 reductase, which utilizes O_2_ and NADPH [49,50]. 

### 2.2. Reduction of Exogenous Flavonoids by Fusaria

The amounts of exogenous flavonoids (400 and 800 μg/g) were significantly reduced in fungal treated cultures, as compared to uninoculated YES + flavonoid controls (Supplementary File S1). This decrease was more evident in samples treated with increased levels (800 μg/g) of flavonoids. Naringenin, the precursor of all flavonoids was efficiently converted by fungal strains to apigenin, luteolin, kaempferol and quercetin. Similar results were obtained by Leonard et al. [49], who found that exogenous supplementation of naringenin increase kaempferol and quercetin amounts in culture media of *Escherichia coli*. The absence of naringenin, as the first precursor of flavonoid biosynthesis pathway in flavone and flavonol treated samples, may indicate inhibition of chalcone synthase activity by these compounds. Treatment of fungi with apigenin led to an increase of luteolin, kaempferol and quercetin in the media, indicating its further efficient conversion. Corresponding results have been found in samples treated with luteolin and kaempferol. However, it was found that besides the formation of the products resulting from flavonoid conversion, fungi treated with luteolin, kaempferol and quercetin accumulated increased levels of their parent metabolites. Luteolin-treated samples exhibited increased levels of apigenin, while in kaempferol-treated samples increased amounts of both flavones (luteolin and apigenin) were found. Unexpected formation of flavones and kaempferol was also evident in quercetin-treated samples. The formation of the intermediate parent metabolites in the presence of exogenous flavones and flavonols remains largely unclear. One can hypothesize that the increase of the parent intermediates could result from insufficient conversion of fungal-derived apigenin, luteolin and kaempferol, which could be produced by fungi at different stages of incubation. On the other hand, the absence of fungal-derived naringenin does not suggest that the production of these compounds occurred in the presence of flavones and flavonols.

### 2.3. Exogenous Flavonoids Affect Production of Phenolic Acids by Fusaria 

The sum of fungal-derived phenolic acids decreased in flavonoid treated samples, however, this effect appeared to be dependent on the strain, flavonoid molecule and its concentration (Supplementary File S1). Among studied phenolic acids, chlorogenic and sinapic acid did not accumulate in the presence of flavonoids, while *p*-coumaric, caffeic, ferulic and syringic acid could be detected in samples treated with naringenin and apigenin. Higher diversity and quantity of phenolic acids in naringenin and apigenin-treated samples could be explained by their relatively lower antioxidant activities (Table 1). Interestingly, the accumulation of *trans*-cinnamic acid in luteolin, kaempferol and quercetin-treated samples suggests their inhibitory effect on converting this first intermediate of the shikimate pathway further.

### 2.4. Effect of Flavonoids on Trichothecene Accumulation in the Media

The effect of flavonoids on mycotoxin production was evaluated by quantification of trichothecenes in treated versus non-treated plates (YES + fungal controls). As typical for solid media [53], the strains co-produced trichothecene compounds at different levels. In addition, all strains tested co-produced lower amounts of trichothecenes not characteristic for their chemotypes (Supplementary File S2). The decrease in mycotoxin accumulation was revealed in most flavonoid-treated plates. However, this effect appears to be largely dependent on the strain, the flavonoid compound and its concentration and appears to be dependent on the antioxidant and antiradical capacity of the assayed flavonoid. The most dramatic reduction of trichothecene accumulation was reported for quercetin. This flavonoid showed the highest antioxidant and antiradical properties compared to the remaining flavonoids (Table 1). The reduction of trichothecene accumulation by quercetin ranged from 78.2 to 99.8%. A lower reduction of mycotoxin accumulation was found in plates treated by both luteolin and kaempferol. The former compound shows slightly higher antioxidant activity than the latter (Table 1). Luteolin caused 49.6 to 99.8% reduction of mycotoxin accumulation, while the inhibitory effect of kaempferol ranged from 24.2 to 99%. 

Naringenin displayed reduction of trichothecene accumulation ranging from 40.9 to 99.7%, except of one strain (MUCL 53469), where this reduction ranged from 10.2 to 40.3%. The inhibiting effect of naringenin is close to results obtained for luteolin, which is rather unexpected when comparing their antioxidant properties (Table 1). It is hypothesized that a high reduction of mycotoxin content by naringenin might result from early conversion of naringenin to luteolin, kaempferol and quercetin by fungi (Supplementary File S1), which more effectively interfere with mycotoxin production. However, high reduction of mycotoxin content by naringenin may be explained by increased growth inhibition reported during the early stage of incubation of fungi on the media (Supplementary File S2). The initial stage of fungal incubation appears to be critical for the total mycotoxin accumulation in the media due to the early activation and high expression of *Tri* genes in the first days of fungal incubation [54]. Changes in growth rate could also partially explain the observed weak inhibition of mycotoxin production in apigenin treated plates. It was found that fungal growth was significantly stimulated in 3 of 12 samples treated with apigenin. In 2 of 3 growth-stimulated samples (CBS 173.31 treated with 400 and 800 μg/g) a 13.7 to 289.4% increase in the sum of trichothecenes was found, as compared to YES + fungal control. 

It is intriguing that two structurally similar compounds (apigenin and naringenin) exhibited quite opposite effects on fungal growth. Apigenin derives from naringenin and differs from the latter by forming a double bond in ring C of the flavonoid structure [47,48] (Table 1). To date, information from literature on mechanisms underlying different biological activities of these flavonoids has been mainly limited to describing their action on human targets [55]. Studies of Zhang et al. [56] showed that this single bond is responsible for large differences in binding affinities of these two flavonoid compounds. It has been demonstrated that single bond rendered naringenin is more flexible to bind to human serum transferrin (Tf) (a single-chain iron-binding blood plasma glycoprotein) [56]. It is hypothesized that the increased binding affinity of naringenin to specific fungal targets may explain its observed higher growth inhibitory effect on fungi. The link between fungal growth changes and mycotoxin accumulation could not be confirmed based on luteolin and kaempferol treated cultures where, in all 7 growth-stimulated samples, a decrease in mycotoxin was reported. The weakest inhibiting effect of apigenin on mycotoxin production is, however, in line with its lowest antioxidant and antiradical properties as compared to other flavonoids tested (Table 1). 

### 2.5. Effect of Flavonoids on the Expression of Tri Genes

The effect of flavonoids on mycotoxin production was tested at the transcriptional level by quantification of *Tri4*, *Tri5* and *Tri10* transcripts on day 3 of fungal incubation. Gene expression results are expressed as the relative transcript levels (Supplementary File S2). 

Desjardins et al. [39] and Takahashi-Ando et al. [40] provided evidence that flavones are inhibitors of cytochrome P450 monooxygenase responsible for the oxygenations of trichodiene at early stages of trichothecene biosynthesis. P450 monooxygenase is encoded by *Tri4*, whose expression, as shown in this study, was drastically reduced in 29 of 60 flavonoid-treated samples. The current results also showed that flavonoids not only inhibit expression of *Tri4*, but also *Tri5* gene encoding trichodiene synthase, a terpene cyclase which converts farnesyl pyrophosphate to trichodiene, the hydrocarbon precursor of trichothecenes [57]. Inhibition of *Tri5* expression might be explained by suppressed expression of the *Tri10* gene, which coordinates expression of four trichothecene pathway-specific genes (*Tri4*, *Tri5*, *Tri6*, and *Tri101*) and the isoprenoid biosynthetic gene for farnesyl pyrophosphate synthetase (FPPS) [58]. However, it should be noted that inhibition of *Tri5* activity occurred in most, but not all, samples with reduced expression of *Tri10* (Supplementary File S2). Except for kaempferol treated samples, the effect of flavonoids on *Tri* gene expression corresponds to the observed differences in mycotoxin profiles of the studied samples. 

Among the tested flavonoids, quercetin (with the relatively highest antioxidant and antiradical properties) showed the strongest decrease of *Tri* transcript levels, in some cases leading to the complete inhibition of *Tri* gene expression. Both luteolin and naringenin showed strong inhibitory effect on *Tri* gene activity, although their effects were largely dependent on the strain assayed. Among the tested compounds, apigenin exhibiting the lowest antioxidant and antiradical properties displayed the weakest inhibitory effect on mycotoxin production at a transcriptional level. Significant inhibition of *Tri* gene expression was found in only 4 of 12 apigenin-treated samples (Supplementary File S2). 

Surprisingly, the effect of kaempferol on *Tri* gene expression was not evident for all studied samples, which is in contrast with its high reduction of trichothecene accumulation in the media (Supplementary File S2). The above result may suggest a different mechanism of action of this compound. Indeed, in contrast to the remaining compounds, kaempferol, has been found to be a more potent hydroxyl than the superoxide radical scavenger [59]. It is hypothesized that the pronounced ability of kaempferol to decrease hydroxyl radical concentration may play a key role in its different activity towards fungal targets. The diverse mechanisms of action of flavonoids have been previously highlighted in Treutter [14,15], who suggested that these compounds might target different components and functions of fungal cells. Such targeting, including binding to enzymes and selected proteins and/or chelation of metals necessary for enzyme activity, as shown in this study, dramatically affects mycotoxin production by fungi with no significant impact on the activity of *Tri* genes.

In this study, it was found for the first time that flavonoids display variable effect on fungal secondary metabolic profiles. The observed high variability is in line with previous reports demonstrating variable effects of other plant resistance related compounds such as phenolic acids [44,45] and lignans [60] on this group of toxigenic fungi. Notably, the in vitro results presented in this paper are in line with recent in planta studies demonstrating that resistance to *Fusarium* diseases is closely associated with the promoted biosynthesis of phenylpropanoids, including flavonoids [33,43,61,62]. It is believed that the results obtained in this study may be significant in developing novel strategies to limit mycotoxins contamination of food and feed.

## 3. Materials and Methods

### 3.1. Fungal Strains

The six fungal strains used in this study (Table 2) are maintained in international fungal collections: Westerdijk Fungal Biodiversity Institute, Utrecht, the Netherlands, MUCL—MUCL Mycothèque de l’Université catholique de Louvain, Louvain-la-Neuve, Belgium and ARS Culture Collection, USDA, Peoria, IL, US. Detailed characteristics of the fungal strains are provided in the ToxGen database [63].

### 3.2. Medium and Culture Conditions

Flavonoid aglycones (Sigma–Aldrich, Saint Louis, MO, USA): Naringenin, apigenin, luteolin, kaempferol and quercetin were dissolved in 10 mL of 96% ethanol and then added to the YES medium to obtain the final concentrations: 400 μg/g and 800 μg/g. The flavonoid concentrations used in this study are close to the amounts produced by plants in the response of *Fusarium* infection [43]. The flavonoids used in this study exhibited different antioxidant and antiradical capacities, which are apparently defined by their structural characteristics [52] (Table 1).

The study incorporated three different controls: YES-only control (YES medium only), YES + flavonoid controls (YES medium supplemented with either 400 or 800 μg/g of flavonoids) and six YES + fungal controls (fungal strains incubated on YES media). The control samples were supplemented with an identical volume of 96% ethanol. Petri plates (Ø 80 mm) were inoculated from 6–8-week old laboratory stock cultures maintained at 4 °C on PDA slants and incubated at 25 °C (in triplicate) in the dark. For gene expression analysis, plates were incubated for 3 days for each condition, while for chemical analysis (mycotoxin, flavonoid and phenolic acid determination) the plates were incubated for 21 days. Growth inhibition tests were performed at an early stage of the incubation of fungi on PDA medium (till the 6th day after inoculation) as previously described by Ponts et al. [64] and Kulik et al. [60].

### 3.3. Determination of Flavonoids and Phenolic Acids in the Medium

0.20 g samples for analyses were weighed and placed in sealed 17 mL culture test tubes, where alkaline and acid hydrolysis were conducted. To conduct alkaline hydrolysis, 1 mL of distilled water and 4 mL 2 M aqueous sodium hydroxide was added to the test tubes. Tightly-sealed test tubes were heated in a water bath at 95 °C for 30 min. After cooling (approx. 20 min), the test tubes were neutralized with 2 mL 6 M aqueous hydrochloric acid solution (pH = 2). The samples were then cooled in water with ice. Flavonoids were extracted from the inorganic phase using diethyl ether (2 × 2 mL). The formed ether extracts were continuously transferred to 8 mL vials and acid hydrolysis was then conducted. For this purpose, the aqueous phase was supplemented with a 3 mL 6 M aqueous hydrochloric acid solution. Tightly-sealed test tubes were heated in a water bath at 95 °C for 30 min. After being cooled in water with ice, the samples were extracted with diethyl ether (2 × 2 mL). The produced ether extracts were continuously transferred to 8 mL vials, after which they were evaporated to dryness in a stream of nitrogen. The samples were dissolved in 1 mL of methanol prior to analyses. The analysis was performed using an Aquity H class UPLC system equipped with an Waters Acquity PDA detector (Waters, Milford, MA, USA). Chromatographic separation was performed on an Acquity UPLC^®^ BEH C18 column (100 mm × 2.1 mm, particle size 1.7 μm) (Waters, Milford, MA, USA). The elution was carried out in a gradient using the following mobile phase composition: A: acetonitryl with 0.1% formic acid, B: 1% aqueous formic acid mixture (pH = 2). The concentrations of flavonoids were determined using an internal standard at wavelengths λ = 320 nm. Compounds were identified based on a comparison of retention time of the analyzed peak with the retention time of the standard and by adding a specific amount of the standard to the analyzed samples and a repeated analysis. The detection level was 1 μg/g. The retention times of the assayed acids are as follows: kaempferol 6.11 min, luteolin 11.89 min, apigenin 16.43 min, chlorogenic acid 21.56 min, caffeic acid 26.19 min, syringic acid 28.05 min, naringenin 31.22 min, quercetin 39.58 min, *p*-coumaric acid 40.20 min, ferulic acid 46.20 min, sinapic acid 48.00 min and *trans*-cinnamic acid 52.40 min, respectively. Fungal-derived phenolic compounds were determined in dried YES-only control, YES + flavonoid controls, six YES + fungal controls and treated fungal cultures after a 21-day incubation period (Supplementary File S1) as previously described in Kulik et al. [45]. 

### 3.4. Determination of the Antioxidant Capacity (VCEAC/L) and Radical Scavenging Activity (ABTS^+^) of Flavonoids

VCEAC/L and ABTS^+^ assays of flavonoids were performed as previously described by Kim et al. [65] and Re et al. [66], respectively. For ABTS^+^ generation from ABTS salt, 3 mM of K_2_S_2_O_8_ was reacted with 8 mM ABTS salt in distilled, deionized water for 16 h at room temperature in the dark. The ABTS^+^ solution was then diluted with a pH 7.4 phosphate buffer solution containing 150 mM NaCl (PBS) to obtain an initial absorbance of 1.5 at 730 nm. Fresh ABTS^+^ solution was prepared for each analysis. The reaction kinetics were determined over a 2 h period with readings every 15 min. Reactions were complete in 30 min. Samples and standards (100 ím) were reacted with the ABTS^+^ solution (2900 µm) for 30 min. Trolox was used as a standard. The results were also expressed as mg Vitamin C equivalent antioxidant capacity per liter (mg VCEAC/L).

### 3.5. Analysis of Trichothecenes from Fungal Cultures

Trichothecenes were determined in fungal cultures treated and non-treated (YES + fungal controls) with different flavonoids by GC-MS as previously described by Perkowski et al. [67].

### 3.6. Extraction of Total RNA and Preparation of cDNA

The total RNA was extracted from 3-day-old fungal cultures from mycelium grown on YES medium treated and non-treated (YES + fungal controls) with flavonoids. Six biological replications were prepared for each condition. Extraction of RNA and reverse-transcription were performed as previously described in Kulik et al. [60,68]. cDNA samples were stored at −25 °C for transcript quantification.

### 3.7. Gene Expression Analysis 

*Tri4* and *Tri5* genes which are responsible for the initial stage in the trichothecene biosynthetic pathway were chosen for qPCR analysis, as previously described in Kulik et al. [60,68]. The analysis also included the *Tri10* gene responsible for regulation of multiple *Tri* genes [58]. Gene expression analyses were performed as previously described in Kulik et al. [44,45]. The Ct values of the target *Tri4*, *Tri5*, *Tri10* and reference *Ef1α* gene were compared in the control and treated samples and normalized relative to the Ct values obtained for the reference *EF1α* gene using the REST 2009 software [69].

### 3.8. Statistical Analyses

The significance of differences among quantities of flavonoids, phenolic acids and mycotoxins and fungal growth was tested using t-Student test at *p* < 0.05. The t-Student test was also used to evaluate differences in the growth of fungi in flavonoid-treated plates.

## Figures and Tables

**Table 1:** A summary of the structures and characteristics of the studied flavonoids.

Subclass	Compound	Substitution at Carbon Position					Double Bond C2-C3	Antioxidant Activity			Antiradical Activity ^2^
		3	5	7	3′	4′		VCEAC/L	ABTS (μM TROLOX/100 g)	Protection Factor 1	(%)
Flavones	Apigenin	-	OH	OH	-	OH	+	190.4	297.5	0.99	0.7
	Luteolin	-	OH	OH	OH	OH	+	483.5	589.7	4.24	n.t.
Flavonols	Kaempferol	OH	OH	OH	-	OH	+	450.7	540.3	2.49	93.5
	Quercetin	OH	OH	OH	OH	OH	+	692.5	744.9	11.50	89.8
Flavanones	Naringenin	-	OH	OH	-	OH	-	301.7	529.4	1.09	6.3

^1^—Antioxidant activity of flavonoids measured in lard oil by a Rancimat test (from Yang et al. [51]). ^2^—Antiradical activities of flavonoids (3.3 × 10^−5^ M) in a methanol solution of DPPH (1.6 × 10^−5^ M) (from Burda and Oleszek [52]).

**Table 2:** List of fungal isolates used in this study.

Species	Strain	Trichothecene Genotype	Origin, Host and Year of Isolation
*F. culmorum*	CBS 173.31, NRRL 26853	3ADON	Canada, oat, 1927
	MUCL 53469	3ADON	Belgium, corn, 2007
	CBS 139512	NIV	Poland, wheat, 2003
*F. graminearum* s.s.	CBS 119173, NRRL 38369	3ADON	USA, Louisiana, wheat, 2005
	CBS 138561	15ADON	Poland, wheat, 2010
	MUCL 53455	NIV	Belgium, corn, 2007

## Supplementary Materials

The following are available online at http://www.mdpi.com/2072-6651/10/3/110/s1. Supplementary File S1. Flavonoid and phenolic acid accumulation by fungal strains after 21 days of incubation of fungi in the presence of exogenous flavonoids. Supplementary File S2. RQ (relative quantification) of *Tri4*, *Tri5* and *Tri10* transcripts and trichothecene accumulation by fungal strains incubated in the response of different flavonoids.
